# Evolution and diversity of the angiosperm anther: trends in function and development

**DOI:** 10.1007/s00497-021-00416-1

**Published:** 2021-06-26

**Authors:** Johanna Åstrand, Christopher Knight, Jordan Robson, Behzad Talle, Zoe A. Wilson

**Affiliations:** School of Biosciences, University of Nottingham, Sutton Bonington Campus, Loughborough, Leicestershire LE12 5RD UK

**Keywords:** Anther evolution, Pollen development, Anther dehiscence, Microsporogenesis, Anther wall formation

## Abstract

**Key message:**

Anther development and dehiscence is considered from an evolutionary perspective to identify drivers for differentiation, functional conservation and to identify key questions for future male reproduction research.

**Abstract:**

Development of viable pollen and its timely release from the anther are essential for fertilisation of angiosperm flowers. The formation and subsequent dehiscence of the anther are under tight regulatory control, and these processes are remarkably conserved throughout the diverse families of the angiosperm clade. Anther development is a complex process, which requires timely formation and communication between the multiple somatic anther cell layers (the epidermis, endothecium, middle layer and tapetum) and the developing pollen. These layers go through regulated development and selective degeneration to facilitate the formation and ultimate release of the pollen grains. Insight into the evolution and divergence of anther development and dehiscence, especially between monocots and dicots, is driving greater understanding of the male reproductive process and increased, resilient crop yields. This review focuses on anther structure from an evolutionary perspective by highlighting their diversity across plant species. We summarise new findings that illustrate the complexities of anther development and evaluate how they challenge established models of anther form and function, and how they may help to deliver future sustainable crop yields.

## Introduction

The anther is the pollen producing part of the stamen, which is supported by a stalk-like filament, and together, they make up the male reproductive structure of the angiosperm flower. Throughout the evolution of the angiosperms, the stamen has undergone a variety of adaptations to enhance reproductive success (Doyle 2012; Endress and Doyle 2009; Sauquet et al. 2017). The range of complexity in anther wall formation, filament attachment and anther dehiscence emphasise the scale of anther adaptability (Endress 1996). The differentiation and formation of the anther walls enclosing the developing microsporangia are still poorly understood across a wide range of angiosperm families. Generally, the anther wall comprises the tapetum, the middle layer and the endothecium which are enclosed by the epidermis (Gómez et al. 2015). The innermost tapetum layer is perhaps the most studied due to its crucial role in microspore development (Cigan et al. 2001; Feng and Dickinson 2010; Furness and Rudall 1998, 2001; Parish 2012; Wilson and Zhang 2009). It is adjacent to the developing pollen, providing materials for pollen wall formation and coordinating the progression of pollen development. There is growing understanding of the gene networks involved in tapetum differentiation and pollen wall synthesis, and evidence of conservation of these networks across monocot and dicots species (Callens et al. 2018; Drábková and Honys 2017; Gómez et al. 2015; Silva et al. 2016; Theissen and Melzer 2007). This highly regulated process is essential for viable pollen production and male fertility, but despite its importance there are still many unresolved questions regarding the origin, function and communication between the different tissues within the anther. In this review, we explore the conservation and divergence of the anther cell layers to provide an overview of their role in reproductive success. We focus on the complexities of the male reproductive system and evaluate the functions of anther structure and the conservation of the mechanisms behind pollen production and release, and the associated drivers of reproductive success.

### Anther development in angiosperms

Since the first appearance of flowering plants, the angiosperms have become the dominant group of land plants (Bell et al. 2010). The occurrence of the most recent common ancestor (MRCA) of the angiosperms has long been of interest, but has proved difficult to accurately place in an evolutionary timeline. Rapid angiosperm expansion occurred during the Cretaceous period about 65–145 million years ago (Ma) (Doyle 2012; Coiro et al. 2019), possibly with multiple radiation events occurring during this period (Bell et al. 2010). Variability in predictions of the molecular clock and lack of fossil evidence of transitory species add to the difficulty of determining the timing of the MRCA (Barba-Montoya et al. 2018; Bell et al. 2010; Coiro et al. 2019; Murat et al. 2017). The discrepancy between fossil evidence and current molecular models is further complicated by differences in molecular evolution between angiosperms and gymnosperms (De La Torre et al. 2017). Overall angiosperms have a molecular evolution rate that is seven times higher compared to gymnosperms making phylogenetic comparisons more complex (De La Torre, et al 2017). The speed and diversification during the early radiation events of the angiosperms further complicate the dating of the MRCA (Li et al. 2019).

A reconstruction of the anatomy of the ancestral flower suggests the MRCA had ten stamens with introrse (inward-facing) anthers, arranged in a whorl and separated from other floral organs (Sauquet et al. 2017). This model, based on floral traits across species, has sparked debate regarding the configuration of the angiosperm MRCA flower (De-Paula et al. 2018; Rümpler and Theißen 2019; Sokoloff et al. 2018). Despite the controversy regarding the exact anatomy of the first flower structure, anther ontogeny has remained largely unchanged within the angiosperm family (Endress and Doyle 2009).

**Fig. 1:**
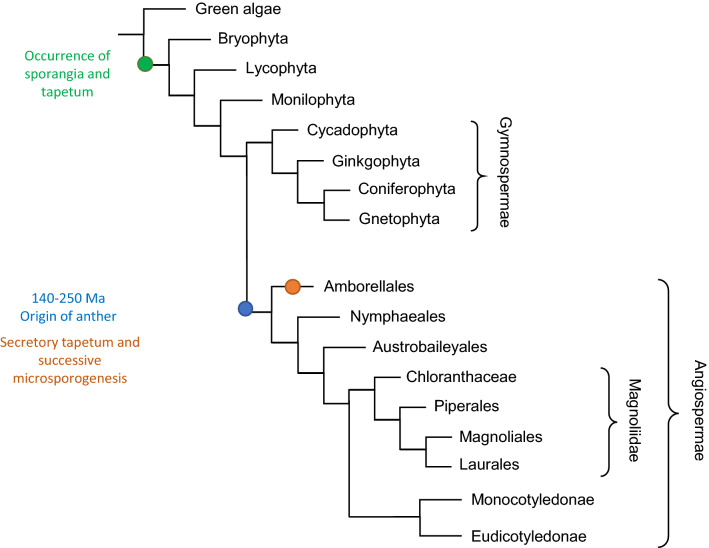
Phylogenetic tree of land plant evolution. The first angiosperm anther is believed to have occurred 140–250 Ma with the advent of the angiosperm flower (Sauquet et al. 2017). Adapted from (Bhattacharya and Medlin 1998; Endress and Doyle 2009)

To address trends in evolution of the angiosperm flower and to put developmental events in perspective, comparisons are often made to living basal angiosperms. The earliest diverging angiosperm is *Amborella trichopoda,* the only member of the Amborellales order, that various reports have concluded is the sister group of all living angiosperms (Fig. 1) (Amborella Genome Project 2013; Burleigh et al. 2011; Jansen et al. 2007). Phylogenetic studies where eudicots and monocots are compared to the *Amborella* genome suggest that most of the evolutionary changes in gene networks are shared between closely related taxa (e.g. Solanaceae family), or in terminal branches of the tree (Amborella Genome Project 2013).

Despite complications in determining the MRCA, there is agreement over the initial molecular steps in male reproduction due to the high level of conservation of fundamental stamen development genes within the angiosperm clade (Callens, et al. 2018; Doyle 2012, Theissen and Melzer 2007). Floral development within the angiosperm clade varies extensively, with differences in organisation, number and type of floral organs. Flowers in *Arabidopsis* develop as four whorls of the floral meristem with the outermost, whorl 1, forming sepals, whorl 2 forming petals, whorl 3 stamens and the innermost whorl 4 forming the carpel (Coen and Meyerowitz 1991). The development and distinctions of these whorls are regulated by classes of genes summarised in the ABCDE model of organ formation. In this model, each class encompasses MADS-box transcription factors that are required for a specific organ differentiation, where the B and C genes together with the E genes, generate stamens (Rijpkema et al. 2010; Theißen and Saedler 2001).

Despite differences in flower development, the MADS-box transcription factors described in the ABCDE model are highly conserved among flowering plants (Callens et al. 2018 and references therein). Molecular models suggest the BC genes responsible for reproductive organ formation predate angiosperms and gymnosperm separation and probably existed 300 Ma (Theissen and Melzer 2007). Despite this timescale, orthologues of the B class genes isolated from the gymnosperm *Gnetum gnemon* can partially rescue B class mutants in *Arabidopsis*, supporting a high degree of functional conservation of the MADS-box genes across the plant kingdom (Winter et al. 2002).

### Diversity of stamen function and organisation

The fundamental role of the stamen is to produce and release viable pollen; however, stamen diversification has led to additional functions within the angiosperm clade. Stamen reduction, or repurposing, has occurred many times within angiosperm evolution and has been used to prevent self-pollination, trigger pollinator–stamen contact, protect the ovary from damage and attract pollinators, via intense coloration and the production of nectar (Walker-Larsen and Harder 2000 and references therein). The diversification of stamens within the same flower, heteranthery, is thought to serve to attract pollinators and enable specific pollinator targeting by sacrificing so-called feeding anthers without impacting overall pollen production (Vallejo-MarÍn et al. 2009). However, recently this view has been challenged by the fact that feeder anthers appear to produce viable pollen and can serve to enhance pollination success by prolonging the timing of pollen release (Kay et al. 2020). This questions this “division of labour” hypothesis and highlights the adaptability and redundancy of the anthers to maximise pollination success (Kay et al. 2020).

In addition to the diversity of stamen function in the angiosperm flower, there is also significant variation in the organisation of stamens. The number of stamens per flower and the type of stamen attachments differ greatly, and these traits are often used as a taxonomic tool for identification of plant families. Stamen filaments also vary, in some basal taxa (e.g. *Amborella* and some Austrobaileyales) these are flattened sporophyll-like organs with abaxial–adaxial polarity, and this contrasts with the radially symmetrical filaments that are typical of more recently emerged angiosperm lineages (Buzgo et al. 2004; Endress 2001a, b; Endress 1996). Nevertheless, the development and the overall structure of the anther has remained remarkably conserved across the angiosperm clade (Doyle 2012; Endress 2001a, b; Hufford and Endress 1989), with divergence in structure and function focusing towards traits that enhance reproductive success (Endress 1996).

### Formation of anther structures from the floral meristem

**Fig. 2:**
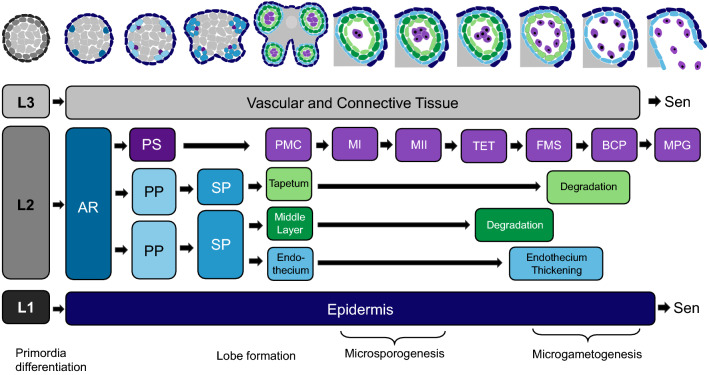
Schematic view of development of anther layers and microsporogenesis in *Arabidopsis*. Stamen primordia differentiate into three cell types, L1, L2 and L3, which further differentiate into the epidermis, the archesporial cells and vascular and connective tissues, respectively. Development of the tapetum, middle layer and endothecium follow the dicot model. AR: archesporial cells, BCP: bicellular pollen, FMS: free microspore, MI: meiosis I, MII: meiosis II, MPG: mature pollen grain, PMC: pollen mother cell, PP: primary parietal, PS: primary sporogenous, Sen: senescence, SP: secondary parietal, TET: tetrad

The development of the stamen is initiated with the differentiation of the floral primordia in the third whorl. The stamen primordia differentiate early into the filament and the anther, with loss of any of the B and C homeotic genes preventing stamen formation (Coen and Meyerowitz 1991). The meristematic cells divide and differentiate to form the reproductive microsporocytes [pollen mother cells (PMCs)] that give rise to the male gametophyte (pollen) and the somatic cell layers that form the surrounding maternal anther walls (Fig. 2).

The meristematic cells consist of three germ layers designated L1, L2 and L3 which differentiate to form the anther (Gómez et al. 2015). L1 gives rise to the outermost epidermis and the stomium cell cluster; L2 gives rise to archesporial cells; and L3 gives rise to the connective tissue, vascular bundle and the circular cell cluster (Schnittger et al. 1996). Specification of the anther is initiated by periclinal division of the L2 layer to form archesporial (AR) cells. The AR cells divide to form two cell layers: the reproductive primary sporogenous (PS) cells that later become the microspores and the somatic primary parietal (PP) cells that differentiate into secondary parietal (SP) cells (Canales et al. 2002). These SP cells divide to form the non-reproductive anther wall layers: endothecium, middle layer and tapetum (Scott et al. 2004; Zhang and Yang, 2014). All anther wall layers except the epidermis are derived from L2 cells (Kelliher and Walbot 2011). Timely formation and degeneration of the various anther layers are essential for viable pollen production. Each layer provides vital functions for microspore development or subsequent pollen release, with failure to form or degenerate at the correct developmental stage leading to male sterility. This is particularly evident for defects associated with the tapetum, which is seen in both cytoplasmic male sterile and genic male sterile mutants used in hybrid plant breeding (Kaul 1988).

### Differential formation of anther layers

**Fig. 3:**
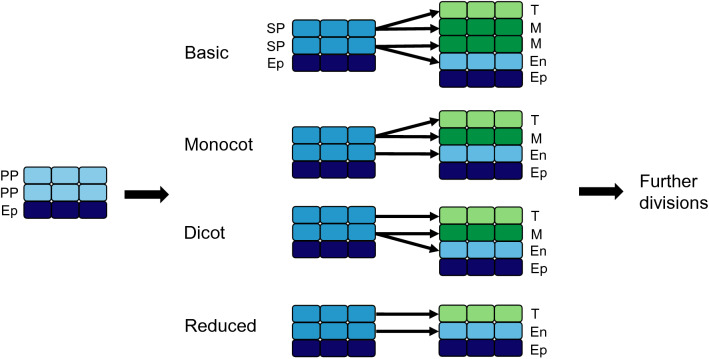
Anther wall formation types (adapted from Davis 1966). In all formation types, the epidermis (dark blue) surrounds the primary parietal cells that differentiate to form secondary parietal cells. The SP cells then differentiate into the endothecium (light blue), middle layer (dark green) and tapetum (light green), according to the formation type associated with each species. Ep: epidermis, En: endothecium, M: middle layer, PP: primary sporogenous cells, SP: secondary sporogenous cells, T: tapetum

The anther wall layers develop through division of the SP layers in one of four types: basic, dicotyledonous, monocotyledonous and reduced (Fig. 3) (Kelliher et al. 2014). It appears that all types follow the same pattern of stamen primordia differentiation until the development of the SP layers, after which the division of these two cell layers determines the formation type. According to Davis (1966) in the most primitive type (basic type), the two SP cell layers divide once each to form four layers: one endothecium, two middle layers and one tapetum. The dicot and the monocot types both result in three layers: one endothecium, one middle layer and one tapetum. The difference is in the origin of these layers, in the dicot model only the outer SP layer divides to form the endothecium and the middle layer, whereas in the monocot model only the inner SP layer divides to form the middle layer and tapetum. In the reduced form, no division occurs, but the two SP layers differentiate to form the endothecium and the tapetum without the middle layer (Davis 1966).

After the differentiation of the cell layers, additional divisions can occur in the endothecium or middle layer to produce extra layers (Carrizo García 2002b). The number of additional layers produced depends on the species and can vary greatly within plant families (Carrizo García 2002b). In members of the Solanaceae family, species forming via the basic type appear to have more variation in the number of middle layers formed, whereas species developing via the dicot formation type rarely formed more than one additional layer (Carrizo García 2002a). In the vast majority of plant species, only one type of anther wall formation is deployed and the same number of wall layers are produced, but there are also examples of two formation types being used within the same anther (Bhandari and Sharma 1987; Hermann and Palser 2000); however, the significance of these differences for pollen development remains unclear.

It has been suggested that the number of cells in the individual anther layers is important to microspore development. Kelliher and Walbot (2011) hypothesise that the middle layer and tapetum form clusters of cells dedicated to the development of specific pollen grains in maize. Derivates of a single SP cell are then earmarked for individual microspores; mutants where the cell layers are abnormal often fail to produce viable pollen (Feng and Dickinson 2010). There is also evidence that the somatic cell layers provide developmental cues to the forming microsporocytes (Wilson and Zhang 2009), including hormone signals regulating anther development (Cecchetti et al. 2017). These observations further support the importance of the correct division, timing and differentiation of SP cells for viable pollen development.

### Anther wall development and function

To a certain extent, the anther cell wall division type is conserved within a phylogenetic group; however, some families display multiple division types suggesting they have evolved several times in angiosperms (Carrizo García 2002b). Anthers in the earliest divergent angiosperm *Amborella*, develop via the “basic type” system (Tobe et al. 2000), whilst the “dicot type” appears to have evolved early in the angiosperm clade in Schisandraceae, in the Austrobaileyales order (Vljayaraghavan and Dhar 1975). In monocots, most members of the Poales order have the monocot type of anther wall development, but some early divergent members have the reduced or the basic type (Sajo et al. 2009). In the Asteraceae, the largest family of the dicots, anthers tend to develop through the dicot type (Ao et al. 2009), whereas in the Solanaceae species, 64% have a basic-type anther and 36% dicot (Carrizo García 2002b). In other families, such as the Ericaceae, several anther types exist (Hermann and Palser 2000), suggesting that the formation type is labile and highly variable throughout the angiosperm clade.

Since the introduction of this system of anther wall classification by Davis (1966), the pattern of cell division of the lineages is generally represented as occurring sequentially in all cells. This view is further supported by studies of anther development where a transverse anther section shows synchronised cells in one plane as representative for the entire anther. There is, however, very little information on the timing of the specification throughout the length of the anther, and the division pattern might not be as rigid as previously thought. When the anther is imaged longitudinally, there seems to be more flexibility in the origin of the cell layers rather than the strict somatic or germinal cell fates depicted in the traditional models (Kelliher, et al. 2014). The cells do not differentiate immediately after divisions, but go through mitosis at different rates making it difficult to determine a single anther wall formation type (Kelliher et al. 2014). Additionally, the classifications offered by Davis (1966) might not encompass all forms of anther wall development. For example, in many species in the Ericaceae the anther wall layers divide in a pattern that cannot be applied to any of the current types (Hermann and Palser 2000), suggesting that the manner of division of the SP cell layers is complex and diverse.

### Evolution of sporogenous cells

Alongside the formation of the anther wall layers from secondary sporogenous cells, the reproductive primary sporogenous cells divide to form the pollen mother cells (PMCs), which go through meiosis to form microspores and subsequently develop into pollen grains. It is in the microsporangia that the microspores divide and develop into mature pollen grains, through the support of the surrounding anther wall layers and the deposition of the intricate pollen wall (Furness et al. 2002). In general, an anther contains four microsporangia divided into two thecae (Endress 1996). The development of sporangia was one of the key innovations which enabled plants to colonise the land, since within these structures hardy spores could be formed which enabled dispersal of genetic material in dry environments (Tanurdzic and Banks 2004).

As a crucial part of the lifecycle of all plants, sporangia exist even in the most basal of land plants: the non-vascular bryophytes (liverworts, hornworts and mosses) and vascular lycophytes (club mosses, spike mosses and whisk ferns) and monilophytes (true ferns). Sporangia have evolved to efficiently disperse spores. In the case of ferns the developing spore mother cells undergo meiosis surrounded by a layer of annulus cells, which dehydrate to cause the sporangia to open at the stomium in a catapult-like manner to rapidly discharge the spores (Noblin et al. 2012). In turn, these spores develop into the gametophytes, which contain antheridia where the sperm cells are produced.

In seed-setting plants, the gametophyte stage is simply condensed into the developing microspore and the mature pollen is essentially the antheridium. Stamens evolved from leaf-like structures bearing microsporangia. Most extant spermatophytes possess stamen-like structures, containing microsporocytes which undergo meiosis to form haploid microspores and subsequently develop into mature pollen grains. The development of pollen from microsporangia is a conserved process for all heterosporous plants including angiosperms and gymnosperms. In terms of thecal organisation, the “stamens” of gymnosperms such as *Gingko biloba*, *Gnetum* and conifers are more simplistic than their angiosperm counterparts. Whereas angiosperm stamens typically have four microsporangia arranged into two theca on each side of the stamen, gymnosperm stamens are disporangiate with a singular microsporangium situated on each side of the filament and thus have no theca (Endress 2001a, b). Whilst there are some angiosperm groups that lack theca (Endress and Stumpf 1990) arguably, organisation of microsporangia into theca was a key innovation in angiosperm anther evolution that has enabled more efficient pollen release.

### Microsporogenesis types

During microsporogenesis, the microsporocytes go through two rounds of meiosis inside the microsporangia to form microspores. Generally, there are two types of microsporogenesis observed, simultaneous and successive, although intermediate types are increasingly being discovered. The types differ in the timing of meiosis in relation to the separation of microspores by the formation of the callose wall. In simultaneous microsporogenesis, meiosis I and II occur without interruption simultaneously alongside callose wall deposition, generally resulting in tetrahedral tetrads. In the successive type there is a pause between meiosis I and II where the callose wall is deposited to form distinct dyads, typically producing tetragonal tetrads (Sajo et al. 2009). However, Furness et al. (2002) argue that the types of microsporogenesis are more complicated than just the simultaneous and the successive, and that the intermediate type is more common than previously thought. Fossil records from the Ordovician period (444–489 Ma) have identified the simultaneous type, prior to the appearance of angiosperms (Furness et al. 2002). In the earliest divergent species from the angiosperms, the Amborellales (Tobe et al. 2000) and members of the Nymphaeales (Taylor and Osborn 2006), the microsporogenesis is successive, suggesting the first angiosperm anther developed via the successive type of microsporogenesis. Most eudicots have simultaneous microsporogenesis with few exceptions (Furness et al. 2002), whereas in the monocots the successive type is predominant, but there are several families where the simultaneous is seen. Taken together, this suggests there have been multiple events of either secondary loss or divergent evolution of the simultaneous and successive types, and a high degree of specificity is preferred in the microsporogenesis type (Furness et al. 2002). However, the significance of this in relation to pollen function and resilience is currently unclear.

### Evolution of tapetum formation and function

The tapetum is the innermost layer of the anther walls (Fig. 2), which is critical for the regulation of pollen development and the synthesis of the pollen wall. Tapetal cells go through a regulated Programmed Cell Death (PCD) that is required for viable pollen formation, where wall materials, such as carbohydrates, lipidic molecules, sporopollenin precursors and nutrients, are secreted into the locules and the developing microspores (Zhang et al. 2011). This specialised cell layer is essential for microspore nutrition and is present in all land plants from the more basal bryophytes to spermatophytes under various descriptors, such as “nutritive cells” or “spore sac layer cells” (Pacini et al. 1985).

The tapetum is almost exclusively single layered; multilayered tapeta are very rare in angiosperms, and in monocotyledons, it has been reported only in *Abolboda* and *Orectanthe* in the Xyridaceae family (Oriani and Scatena 2015). The number of layers of the anther wall seems to be critical for pollen production, with changes in the tapetum being particularly detrimental, with additional tapetum layers resulting in male sterility (Cecchetti et al. 2015; Chaubal et al. 2000, Feng and Dickinson 2010).

There are generally two types of tapeta. The first is the secretory, or glandular, where the tapetum remains in situ in the anther locule whilst synthesising and secreting pollen wall materials, and subsequently breaks down. The other is the plasmodial, or amoeboid type, where the tapetum cell walls break down to release protoplasts that fuse to form a multinucleate plasmodium. A third, less common, type is the invasive tapetum, which is mostly found in the Asteraceae, where the cell walls of the tapetum dissolve and disperse among the developing microspores (Tiwari and Gunning 1986). Individuals usually have only one type of tapetum, but there are occasions, e.g. in safflower *Carthamus tinctorius*, where two types of tapetal cells coexist (Yeung et al. 2011). Additionally, Sajo et al (2005) hypothesise there might be an intermediate tapetum type, where an early-stage secretory tapetum subsequently becomes invasive. The significance of two types coexisting, working independently or transitioning from one to another is still unclear, but it suggests there are intricate adaptations and specific regulation of tapetum development.

Both secretory and plasmodial types are common in dicots and monocots. The secretory tapetum probably had several independent origins (Oriani and Scatena 2015) and is regarded as the most primitive form (Furness and Rudall 1998; Pacini et al. 1985). The early divergent angiosperm *Amborella* has a secretory tapetum (Tobe et al. 2000), which also appears to be the most common type in primitive dicotyledons (Furness and Rudall 1998). Both types of tapeta are found throughout the monocot clade, suggesting secretory and plasmodial forms have evolved several times in monocotyledons (Furness and Rudall 1998). The variability in tapetum type within the angiosperm clade, where reversals or re-evolving of the types occurs throughout the taxa, again further supports the significance of the tapetum and the importance of precise control of its development and function.

### Variability and function of the anther middle layer in pollen development

The function of the middle layer is not fully established since mutants displaying middle layer defects also tend to show tapetum abnormalities, making it difficult to determine its independent function. The middle layer was long thought to have no function and be a leftover relic from pre-angiosperm anther structures (Davis 1966). More recently, however, it has been shown to have a secretory function similar to the tapetum, with failure in degeneration leading to male sterility through delayed exine deposition (Falasca et al. 2013). Once the middle layer has formed it becomes thinner throughout pollen formation and is completely degenerated by anther dehiscence. Kelliher and Walbot (2011) hypothesise that the tapetum and middle layer derive from a single secondary parietal cell, providing the nutrients required for the development of a single microspore.

The middle layer is important for pollen formation in many species yet there is high variability in the number of middle layers. No middle layer is formed in reduced-type anthers (Fig. 3), whereas some species undergo additional divisions to form multiple layers, with as many as nine middle layers in *Hawkesiophyton panamense* in the Solanaceae family (Carrizo García 2002a). The reason for this diversity in the number of layers, along with the specific function of the middle layer is currently not known, although may be due to environmental responses. In cereal crops, drought stress during the meiotic and mitotic stages can lead to an expanded middle layer, possibly due to increase sugar transport to the microspores in response to water deficiency (Yu et al. 2019). In late stamen development, the middle layer is involved in pollen maturation and anther dehiscence by controlling auxin signalling (Cecchetti et al. 2017), suggesting that the middle layer may be important in signalling during multiple stages of anther and pollen development.

### The importance of anther wall PCD for sex determination

Developmentally programmed cell death (dPCD) is an integral process for pollen development and release, as well as floral organ differentiation (reviewed in Wang et al. 2021). Degeneration of the tapetum and the middle layer provides nutrients to the developing microspore, and degradation of the septum and stomium facilitates anther opening. A common cause of male sterility is the mistimed degeneration of anther wall layers, primarily the tapetum. Mistimed PCD prevents viable pollen formation and is one of the strategies developed by angiosperms to produce unisexual flowers (Flores-Rentería et al. 2013; Hernández-Cruz et al. 2019; Ren et al. 2019). Normal degeneration of the tapetum and middle layer tissues is vital for the provision of nutrients and wall materials for pollen maturation. Infertility in a variety of species such as *Tapiscia sinensis,* an androdioecious tree, or in dioecious cacti, in the *Opuntia* family is ensured by preventing the deposition of essentials materials for mature pollen formation, without the need to restructure the anther (Flores-Rentería et al. 2013; Hernández-Cruz et al. 2019; Ren et al. 2019).

## Anther dehiscence

### Types of anther dehiscence

Across the angiosperm clade, many different types of dehiscence have evolved. The way in which the anther opens is one of many factors that determines the pollination syndrome of a flower (Bernhardt 1996). The dehiscence process is determined by the shape, position and anatomical features of the stomium, endothecium as well as the anther attachment point.

There are four known types of anther splitting: longitudinal, transverse, poricidal and valvate that can occur as introrse (pollen release towards the centre of the flower) or extrorse (pollen release outwards, away from the centre of the flower). In longitudinal dehiscence, the anther splits along the long axis of the theca and is the most common method of anther opening. It is typical of many angiosperms and is found in both monocotyledonous and dicotyledonous species in a wide taxonomic range. Transverse dehiscence is similar to longitudinal dehiscence; however, the split is at right angles to the long axis of the theca. Poricidal dehiscence can be seen in anthers that shed their pollen via terminal apertures, for example in members of the Melastomataceae (Renner 1989), Solanaceae (Bohs 2005) and Leguminosae (Marazzi et al. 2007). Poricidal dehiscence has adaptive value to pollinators capable of collecting pollen by the high frequency vibration of stamens (De Luca and Vallejo-Marín 2013; Larson and Barrett 1999). Finally, valvate dehiscence is where pollen is released through a pore that is covered by a flap of tissue. This type of dehiscence is rare, but is occasionally seen in members of the Hamamelidaceae (Hufford and Endress 1989), Magnoliids (Endress and Hufford 1989) and Berberidaceae (Batygina 2002).

### Endothecium development and secondary thickening

Multiple anther wall layers appear to work collectively not only in the development of the microspores, but in the release of mature pollen grains. The endothecium plays an important role in anther dehiscence and pollen release by interacting with the middle layer and the tapetum. After microspore meiosis, alongside degeneration of the tapetum and middle layers, the endothecium undergoes specific secondary thickening (Wilson et al. 2011). Thickening of the endothecial layer occurs prior to anther dehiscence and serves to build tension in the remaining anther layers to generate sufficient force to break the stomium, retract the anther walls as they dehydrate and disperse the pollen (Keijzer 1987; Nelson et al. 2012). In addition to aiding anther dehiscence, the endothecium appears to serve as a last storage site for lipids during the final stages of pollen development, and fatty acids derived from the endothecium are thought to help facilitate pollen hydration (Zhan et al. 2018; Zhu et al. 2020). Formation of secondary wall thickening in the *Arabidopsis* endothecium layer appears to be regulated principally by three transcription factors *MYB26*, *NAC Secondary Wall Promoting Factor 1* (*NST1*) and *NST2* (Mitsuda et al. 2005; Yang et al. 2007 2017). This secondary thickening process appears conserved across different species and is at least in part orchestrated via auxin signalling (Cecchetti et al. 2013); however, few genes have been isolated that are specific to the endothecium.

Endothecial secondary thickening is highly variable between taxa with distinct deposition patterns impacting anther opening. Attempts have been made to draw phylogenetic information from these patterns, but this has been unsuccessful due to the complex interspecies variation observed (Carrizo García 2002a). Endothecial thickening patterns seem insensitive to ecological influences and there is no direct correlation between them and the dehiscence type (Manning 1996). For example, Manning (1996) highlights that “U-shaped” thickenings occur in different species with each of the different dehiscence types, in anthers that are versatile (filament attached at the centre) or basifixed (filament attached at the base).

Some angiosperm clades do not develop endothecium thickening, but utilise alternative mechanisms to generate the force required to open the stomium. These include some species within the Ericaceae (Hermann and Palser 2000), Leguminosae (Marazzi et al. 2007) and Melastomataceae (Cortez et al. 2014). Most species within the Ericaceae form an alternative specific fibrous tissue called the resorption tissue that has been reported to be involved in anther dehiscence (Hermann and Palser 2000). The Senna group of the Leguminosae rely on thick walled hypodermal and subhypodermal cells as an alternative to the classical thickening of the endothecium (Marazzi et al. 2007). Uniquely, the Melastomataceae represents one of few families with poricidal dehiscence that does not have a specialised mechanical tissue (Cortez et al. 2014). Successful dehiscence in this family relies on specific dehydration of cells in the pore region of the anther, whilst the cuticle prevents dehydration of the surrounding cells (Cortez et al. 2014).

### Anther stomium and septum degeneration

For pollen to be released from the anther, the septum and stomium must undergo controlled degeneration. During anther dehiscence the septum, a region of cells located between the lobes of the theca, degrades to create a single lobe. The stomium is formed by differentiation of epidermal cells along the anther which produces a single cell region through which pollen will be released (Wilson et al. 2011). This differentiation of cells in the epidermis occurs early in development, at a similar time to when the tapetum forms (Bonner and Dickinson 1989).

Solanaceous species have a unique adaptation that is not found in either *Arabidopsis* or *Lilium*. They possess specialised cells in the “notch” region under the stomium called the circular cell cluster (also referred to as: intersporangial septum, hypodermal septum or oxalate package). These highly specialised subepidermal cells, derived from the L2 primordium that accumulate and release calcium oxalate, which is thought to be important for dehiscence and to provide calcium for pollen germination (Iwano et al. 2004). Cells within the circular cell cluster degenerate prior to those in the stomium, facilitating the formation of a bilocular anther. In other non-solanaceous species that lack a circular cell cluster, this role is facilitated by different non-specific cells found in the “notch” region.

### Anther dehydration

Anther dehydration is one of the final processes that facilitate anther dehiscence and pollen release. Firstly, once the pollen grains have fully developed, the locular fluid is removed to facilitate pollen dispersal. Next, the anther wall is dehydrated, which is hypothesised to be crucial to generate the required force to bend and open the anther (Nelson et al. 2012). This process is orchestrated by dehydration of the anther walls causing the anthers to retract (Keijzer 1987). In cereal crops, temperature has been shown to influence anther dehiscence (Fernández-Gómez et al. 2020; Matsui and Hasegawa 2019). In barley, the fertility of *HvMS1* overexpression lines display temperature dependent reversible sterility. Anthers of the *HvMS1* overexpression line produce viable pollen, but fail to dehisce at lower temperature and lead to a reduction in seed set in barley (Fernández-Gómez et al. 2020). Additionally, the duration of anther dehiscence influences seed set in rice, specifically at higher temperatures longer anther dehiscence is seen which favourably improves pollination (Matsui and Hasegawa 2019).

### Impact of hormones on stamen development and pollen release

Most hormones have been shown to be involved in all stages of stamen development, as reviewed by Chandler (2011). Gibberellins have generally been associated with early filament elongation and tapetum development, whereas jasmonic acid (JA) has been linked to later stages of pollen maturation, filament extension and anther dehiscence in higher land plants (Marciniak and Przedniczek 2019; Susheng Song et al. 2011). The effects of hormonal signalling on fertility are not always conserved among land plants. In *Arabidopsis*, MYC proteins promote stamen development by activating JA signalling (Chen et al. 2016), whereas the orthologous MYC proteins in the liverwort *Marchantia polymorpha* do not affect fertility despite being involved with JA signalling (Peñuelas et al. 2019).

Defects in auxin signalling through the disruption of ARF6 and ARF8 function prevents stomium degeneration and pollen release (Nagpal et al. 2005; Zheng et al. 2019). In addition, the function and production of auxin in regard to floral organ initiation have been shown to be conserved in highly diverged plant families, suggesting the hormonal network evolved in an common ancestor (Chandler 2011 and references therein).

Lack of information in basal lineages has made it difficult to confirm the evolution of the auxin pathway in vascular plants. The auxin response is proposed to have emerged as a response to multicellular growth in land plants, although this can be questioned by the presence of auxin pathways in green, red and brown algae (De Smet et al. 2011; Lau et al. 2009; Le Bail et al. 2010; Rensing et al. 2007; Sztein et al. 2000). Identification of a putative functional YUCCA gene, involved in auxin biosynthesis, in green algae suggests some conservation in basal lineages of land plants (De Smet et al. 2011). Furthermore, bryophytes have been shown to have a basic nuclear auxin pathway that contains three classes of ARFs and TIR1/AFB-AUX/IAA co-receptor, indicating the presence of an auxin response pathway in a common ancestor of land plants (Kato et al. 2018; Lavy et al. 2016; Plavskin et al. 2016; Tsuzuki et al. 2016). It is thought that ARF-like transcription factors and auxin co-receptors were established in charophytes, but their involvement in auxin signalling is unknown (Kato et al. 2018; Wang et al. 2015). The role of auxin in the regulation of anther dehiscence has been established in land plants such as *Arabidopsis* (Cecchetti et al. 2013) and rice (Shiyong Song et al. 2018; Zhao et al. 2013). However, better understanding of the evolutionary development of this response in land plants, compared to basal lineages, requires the identification of hormonal response in more diverse species.

## Conclusions

The evolution of the anther was a key event associated with the reproductive success of the angiosperms. The complexity and diversity in the formation, anatomy and dehiscence of the male reproductive organ highlight the importance of the stringent control required for successful pollination. Model species allow detailed study of these phenomena; however, significant diversity exists throughout the angiosperm clade which is important to acknowledge to prevent generalisations about pollination. Studying pollen development and the anther function in an evolutionary context helps facilitate understanding of essential genetic pathways and developmental events, and can be used to further sustainable plant breeding and agricultural practices.
