# Correction to: Evolution and diversity of the angiosperm anther: trends in function and development

**DOI:** 10.1007/s00497-021-00429-w

**Published:** 2021-09-13

**Authors:** Johanna Åstrand, Christopher Knight, Jordan Robson, Behzad Talle, Zoe A. Wilson

**Affiliations:** School of Biosciences, University of Nottingham, Sutton Bonington Campus, Loughborough, Leicestershire LE12 5RD UK

## Correction to: Plant Reproduction https://doi.org/10.1007/s00497-021-00416-1

Unfortunately, the original publication of the article has an error in the labelling on Fig. 3. The correct figure is given in this correction.

**Fig. 3:**
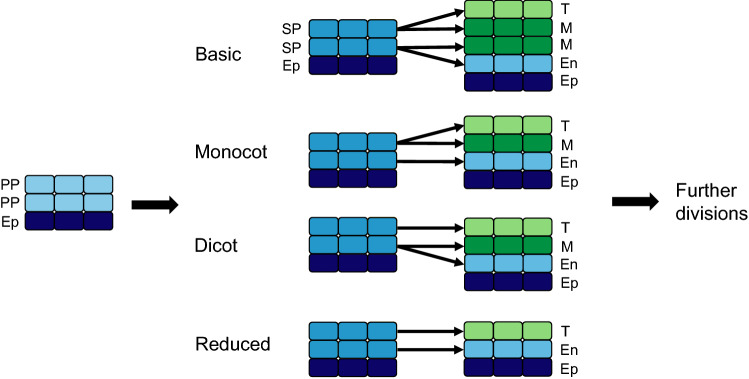
Anther wall formation types (adapted from Davis 1966). In all formation types, the epidermis (dark blue) surrounds the primary parietal cells that differentiate to form secondary parietal cells. The SP cells then differentiate into the endothecium (light blue), middle layer (dark green) and tapetum (light green), according to the formation type associated with each species. Ep: epidermis, En: endothecium, M: middle layer, PP: primary sporogenous cells, SP: secondary sporogenous cells, T: tapetum

The original article has been corrected.

